# P-837. Characteristics, Predisposing Factors And Clinical Outcomes of Patients with Probiotics Organisms Bacteremia

**DOI:** 10.1093/ofid/ofae631.1029

**Published:** 2025-01-29

**Authors:** Hind El Soufi, Santosh Dahal, Thien-Ly Doan, Rubab Sohail, Cristina Sison, Meredith Akerman, Bruce Hirsch

**Affiliations:** Zucker School of Medicine at Hofstra/Northwell, Manhasset, New York; Northwell - Long Island Jewish Medical Center, New Hyde Park, New York; Long Island Jewish Medical Center, New Hyde Park, New York; Northwell Health, New Hyde Park, New York; Feinstein Institutes for Medical Research, Manhasset, New York; Northwell Health, New Hyde Park, New York; Hoftsa Northwell School of Medicine, Manhasset, NY

## Abstract

**Background:**

Bacteremia with probiotic organisms is rare. Patients’ characteristics and clinical outcomes are not well known. Our objective is to study the characteristics, risk factors and outcomes of patients with different probiotic organisms’ bacteremia.

**Figure:**
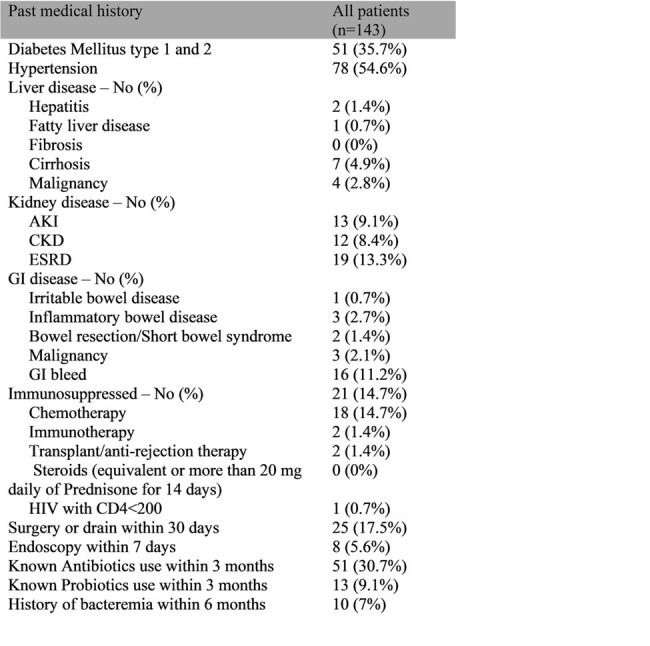

**Methods:**

This is a retrospective observational study of patients with probiotic organisms’ bacteremia, admitted to a New York Health system, between 2018 and 2021. 143 patients were divided into 2 cohorts. Cohort 1 (85.3%) had Lactobacillus spp bacteremia and Cohort 2 (14.7%) had positive blood culture with Saccharomyces spp or Bifidobacterium spp. Descriptive statistics were reported for patients’ characteristics, past medical history (PMH) and hospital related history (HRH). Chi-square or Fisher’s exact test was used for categorical variables and T-test or Wilcoxon test for continuous variables. Logistic regression was used to compare 30-day in hospital mortality and 30-day hospital readmission for a related infection. Negative binomial regression was used to compare hospital length of stay, duration of therapy targeted for probiotic bacteremia and number of days until blood culture clearance. P value < 0.05 was considered statistically significant.

**Figure:**
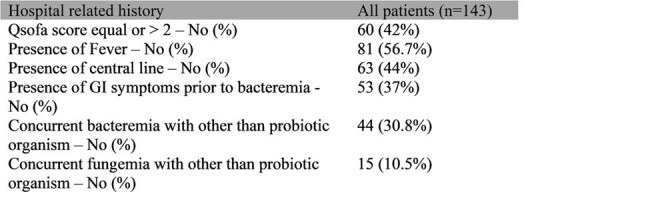

**Results:**

50.3% were male, 59.1 % were white, 2.8 % were Hispanic or Latino. 7% had prior bacteremia within 6 months, 30.8% had concurrent bacteremia and 10.5% had concurrent fungemia with other than the probiotic organism. Patients with probiotic bacteremia (PB) had frequent comorbidities: 48% had hypertension, 36% had Diabetes Mellitus, 14.7% were immunosuppressed. 44% had central line during hospitalization, 42% had qSofa score >2. PB was associated with prolonged length of stay (Mean in days 20.4) and 30-day in hospital mortality (34.3%). There were no statistical differences in PMH, HRH and outcomes between the Lactobacillus spp group and other group.

**Figure:**
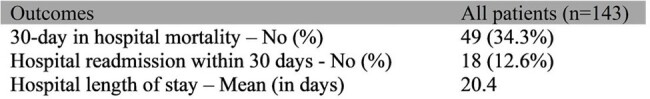

**Conclusion:**

PB occurs in hospitalized patients with frequent comorbidities and advanced clinical illness. Caution is required in administration of probiotics in vulnerable patients.

**Disclosures:**

**All Authors**: No reported disclosures

